# Love, wind eggs, and mere conceptions: non-generation in William Harvey’s *De conceptione*

**DOI:** 10.1080/0950236X.2019.1648100

**Published:** 2019-08-16

**Authors:** Isabel Davis

**Affiliations:** Birkbeck, University of London, UK

**Keywords:** William Harvey, false pregnancy, conception, uterus, wind eggs, psychophysiology

## Abstract

This essay offers a new reading of William Harvey’s *De conceptione*, considering for the first time its interest in non-generative conception. Further, it considers the way that Harvey entangles observations about erotic and maternal love in his discussions of the conceiving body. Love provides a context which Harvey reads for information about conception. But, for Harvey, conception and generation are not synonymous. Conceptions can be without as well as with a foetus, and Harvey is at least as interested in non-generation as he is in generation, and false pregnancy as pregnancy. Harvey’s notion of an immaterial or ‘mere’ conception, on which he builds an intricate analogy about the relation of uterus and brain, is designed to accommodate un-reproductive as well as reproductive experience. Reading signs of love – desires, devotions, intimacies – gives Harvey a way of distinguishing between different kinds of reproductive non-events, health and pathology. He offers an extended consideration of wind eggs and uses fictions of the wind to credit the loves of those that produce no offspring as nonetheless creatively conceiving and biologically demonstrative.

William Harvey’s writing on conception is necessarily caught up with the question of love: both the erotic love which precedes conception and the maternal love emergent in its aftermath. This essay considers how these kinds of love are implicated in Harvey’s understanding of conception. As well as considering forms of human love, my discussion also takes in the cross-species love of a pet – a parrot – for its owner – Harvey’s wife Elizabeth – and the evidence it gave Harvey for understanding the place of sexual love in un-reproductive health and illness. The relationships between love and conception are, in Harvey’s view, very close. In particular, in an odd little treatise, *De conceptione*, which is appended to Harvey’s larger work *Exercitationes de generatione animalium*, he describes an intimate relationship between brain and uterus, an idea of conception which is thoroughly embedded in the ‘psychophysiology’ of his age, which read biology and the passions together.^1^ Some modern scholarly accounts of Harvey’s brain and uterus analogy have dismissed it as ‘failed’ and even ‘ludicrous’, in particular because of its dependence on an error: the impression that there was no material connection between sperm and embryo, and that conception was therefore immaterially produced.^2^ Some, too, have been disappointed that Harvey, so evidently proto-modern in his discovery of the circulation of the blood, did not evade the strictures of history in his work on generation.^3^ However, these dissatisfied critical accounts have overlooked the fact that Harvey’s thinking was informed not just by the question of generation, but also by non-generation, a topic for which immateriality is more germane. In this essay I rehabilitate *De conceptione*, discussing it alongside the larger work *De generatione* to which it is appended, as an articulate account of non-reproductive experience, using the work of feminist Elizabeth A. Wilson on the psychosomatic to listen to Harvey’s ideas anew.^4^ In particular this essay recognises that Harvey’s account of the immateriality of conception gave him a way to understand loves which were without issue, as nonetheless conceiving, image-making, and biologically demonstrative.

First, I lay out the analogy between conceptions in the brain and uterus which principally concerns *De conceptione* before later setting this analogy within a picture of erotic and maternal affections. In *De conceptione*, Harvey speculates on the relationship between the physiological and psychological effects of the sexual act:

the vertue proceeding from the *Male*, doth so largely fructifie the whole *Female*, that it produceth a thorough change and alteration, as well as the frame of their *minds*, as in the constitution of their *bodies*. (541)

The effect of sex, he concludes, is not local to, and does not isolate the sexual or reproductive organs, such as the uterus. Instead, masculine ‘vertue’ (*virtus*), or power, transforms the entire woman, body and mind together. Harvey arrives at this position, on the unity of mind and the female reproductive body, through one of his central observations about animal generation, which he returns to regularly in his work: the absence of any appreciable continuous mass linking sperm and embryo:

since I plainly see that nothing at all doth remaine in the *Uterus* after *coition*, whereunto I might ascribe the *principle* of *generation*; no more then remaines in the *braine* after *sensation*, and *experience*, whereunto the *principle* of *Art* may be reduced. (546)

This material absence or gap was observed through dissection of animals at different temporal points between coitus and the time when an embryo could be seen with the naked eye. These two quotations offer differing, though related propositions. In the second, brain and uterus share only the habit of making copies without direct material connection with their originals; they are thus only rhetorically related. On the other hand, in the first quotation, Harvey suggests a much more integrated account of the female mind and reproductive body. Here, the changes that take place in the uterus after sex are not just similar to the changes that take place in the brain after sensation and experience; instead, sex *is* sensation and experience and it alters the mind at the same time as it effects change in the uterus.

Thus Harvey develops an ambiguous notion of how alike the uterus and brain are; so that at times he brings together the different propositions represented in these two quotations, and at others he holds them further apart. That vacillation about their exact relation can be seen in this passage, in which Harvey lays out his analogy again, but with variation:

and seeing the substance of the *Uterus*, now ready for *Conception*, doth so neerly resemble the Constitution of the Braine: why may we not imagine, that both their functions are also alike; and that something like, if not the self same thing that the *phantasme*, or *appetite* is to the *brain* is excited in the *Uterus*: from which the generation or procreation of the *Egge* doth succeed? For both their functions are equally called *conceptions*, and both are *Immaterial*. (542–3)

Here, Harvey considers the exact relation between conceptions in the brain and the uterus to be uncertain, offering alternatives – ‘something like, if not the self same thing’ (*istuc idem, vel saltem ejus analogum*) (295). Whilst Harvey’s figure is usually understood in modern critical scholarship as an analogy, in fact he never rules out the prospect of these two kinds of conception being not just rhetorical substitutes but being identical, naturally and irrevocably inseparable. In that respect, Harvey prevaricates over what Charis Charalampous has termed ‘the intelligent body’; how far does the body itself think?^5^ Correspondingly, in Harvey’s etymological and rhetorical observation at the end here about the polysemy of *conceptiones*, he does not spell out exactly how the two senses of the word relate and what a shared etymology precisely means in this case. Again, he leaves the nature of the relation undecided.

One aspect of this question about the degree of similarity between the two organs is the rapport between look and function:

First of all the *uterus* appears *thicker* and more *fleshy*: and afterwards (forasmuch as concerneth the *interiour superficies*, which is the place where the future *conception* is to be received) it groweth more *tender*, answering in *lubricity* and *softness* to the internal ventricles of the *Braine*, as we have even now affirmed concerning *Hindes*, and other creatures which *cleave the hoofe*. (542)

Observing the visual and textural similarity between the increasing fleshiness of the endometrium in the later, luteal phase of the mammalian menstrual cycle, and the sponginess of brain tissue, Harvey notes that the uterus becomes more or less brain-like depending on its receptivity to and readiness for conception. Harvey cannot come to a definitive conclusion on the exact proximity between brain and womb because they constantly move together and apart, in rhythmic relation to cyclical change. Time and seasonality are critical factors in Harvey’s understanding of this rhetorical proximity between womb and mind.

Whenever Harvey thinks about seasonal change, springtime coupling and the birds’ broodiness, he turns to Book II of Virgil’s *Georgics*, which attributes this ripening in the natural world to the influence of Zephyrus:

Zephyrique terpentibus auris
Laxant arva sinus, superat tener omnibus humor,
Parturit omnis ager, etc’. (*De generatione*, Latin 38)

[the meadows ungirdle to the Zephyr’s balmy breeze; the tender moisture avails for all, the bounteous earth prepares to give birth].^6^

After this quotation Harvey offers this analysis: ‘[a]nd therefore the people of old, seeing their Hens in the Spring-time lay, this wind then blowing, did conceive Zephyrus to be the Author of the generation of those eggs’ [*Zephyrum eorundem procreationis autorem crediderunt*] (68; Latin 38). The personification of Zephyrus anthropomorphises springtime proliferation, writing it as erotic verse. Harvey recognises that Zephyrus’s authorship is a poetic or folkloric conceit; nonetheless he finds this amatory fiction useful to think through reproductive and sexual maturation, demonstrating what Gail Kern Paster has described as the ‘oscillation of metaphorical and literal comparisons between the wind and the passions’ in early modern culture.^7^ If being alike and being the same thing are on a spectrum of relation, rather than being opposites, moving together and apart in relation to cyclical time, then the brain and uterus’s rhetorical proximity, the distance between fiction and fact, also participates in this shifting natural drama. So, how alike the brain and the uterus are in their conceiving of things depends upon cyclical ripening, just in the same way that hens grow broody in the spring. The womb is ideational and imaginative seasonally and best understood with reference to fictions that embed human sexual love in a wider reproductive ecosystem.

When Harvey thinks about the brain and its making of copies, reproduction in the broadest sense, he thinks about it in two ways. On the one hand, in some parts of his treatise, he makes a particular example of the artistic brain: ‘how the brain of the Artist, or the Artist himself, by virtue of his brain, doth form things which are not present with him, but such as he only hath formerly seen, so much to the life’ (545). On the other hand, at the same time, Harvey makes a special case of the female brain, understanding heterosexual female desire to be instigated by a sensory perception of the external male body, the ‘appetible or desirable object’ (543), which enters the brain through the eye. This cognitive process – in an artist’s or a woman’s brain – is ‘artificial’ or ‘animal conception’ [*conceptus artificialies / animalis*, 295–6], and Harvey speculates that an analogous process operates in the uterus, which he dubs ‘natural conception’ [*conceptus naturalis*], which forms the child in the image of its father. Of ‘natural conception’, Harvey writes: ‘[a]nd from this Appetite or Conception it cometh to pass, that the female doth produce an offspring like the male Genitor’ (543). In these terms, ‘animal conception’ differs from ‘natural conception’ in that it proceeds from cognitive agency, happening in the brain rather than the uterus. Incidentally, elsewhere, Harvey considers how it is that children resemble both parents and ‘must needs be mixt’ (*De generatione* 260–1, see also *De conceptione* 555), but here he is interested in how, without material transmission, the *idea* or form of the father is realised in the offspring. Because there is no appreciable connector, Harvey transfers the authority for ‘natural conception’ to a uterine ‘appetite’ as much as to the masculine *virtus* to which it responds.

In her study of the politics of Harvey’s analogies, Eve Keller rightly argues that Harvey’s writings are ‘filled with sex stories’.^8^ She has also found Harvey to settle primarily on masculine dominance and female passivity quite in the face of the logic of his own findings on the ovum; she describes him ‘taking back his assertions of her [i.e. woman’s] procreative agency’.^9^ Yet, those places at which Harvey departs from Aristotelian authority to write feminine agency in conception remain points of issue in his work, rather than being cancelled as Keller suggests.^10^ Harvey displaced the gendering inherent in traditional accounts of Aristotelian hylomorphism – that matter was feminine and naturally inclined to masculine form – by crediting feminine conception with cognitive forming agency. At the same time he kept the notion of female inclination or desire in his accounts of conception; so feminine desire for a male partner was met by, was similar to or perhaps the self-same thing as a desire within the uterus, both were image forming and potentially reproductive.^11^ Thus Harvey breaks up the neat gender binary which often attended accounts of matter-form relations to come to his notion of the ‘mixt’ conception. In this way ‘animal conceptions’ were as much female as male, and as much the responsibility of women as were ‘natural conceptions’. Indeed, Keller herself concludes that Harvey’s masculine posturing is unconvincing; so, as it turns out, Harvey’s position is an unsettled prevarication which, as James G. Lennox notes, contradicts Aristotle to argue for male and female as co-efficient causes in reproduction.^12^

The relationship Harvey draws between these parallel kinds of conception, natural and animal, bears much in common with the contemporary idea of the maternal imagination, the notion that the foetus could be imprinted with those things that women viewed in pregnancy: their partners, of course, but also images or objects.^13^ As theories of paternal heredity they share an understanding of the mind and the body as very close, and the uterus as image forming. On the other hand, however, Harvey’s notion is different. Indeed, the notion of the maternal imagination mixes up Harvey’s two ideas of conception: the animal and the natural, making animal conception operate through sight rather than through the immaterial virtue of sperm. In the maternal imagination model, instead of the uterus being itself brain-like and intelligent, the uterus is governed by and subordinate to the brain and, so, sense perception. In Harvey’s version, paternal heredity is communicated *as if* by sight and, whilst it is then conceived, or interpreted, inside the female body, that conception is carried out by the uterus rather than the brain. Another difference is that the idea of the maternal imagination was linked particularly to teratology, a way of explaining foetal abnormality as well as paternal resemblance. Harvey is interested in foetal development, and of thinking about heredity in terms of image theory, but the emphasis in *De conceptione* is on the prior conception event. Rather than trying to answer questions about the generation of monsters, Harvey was researching the moment and mechanism of conception, especially given its apparent immateriality.

I return now to that starting point: the lack of material relation, the gap, between sperm and embryo, because it has been important for other critical responses to *De conceptione*. Of course, since Harvey, the gap he observed has been infilled by new discoveries in endocrinology, reproductive physiology, genetics and embryology. Historians of science point out that, because Harvey was looking without the benefit of a microscope, although that technology was available, he missed the material continuity within conception.^14^ As it turns out, there is no gap. Historians have addressed the problems they see predicated on this omission in a number of related ways. Benjamin Goldberg understands Harvey’s analogies as valuable ‘thought-experiments’.^15^ Nonetheless he concludes that the central brain-uterus analogy fails, and, picking up Harvey’s etymological observation about the word *conceptiones* which I cited above, concludes that the analogy ‘now seems a ludicrous theory based upon, at best, homonymy’, a view which places linguistic practice a long way from any ‘truth’ about the body.^16^ Goldberg distances his own reading from that of Guido Giglioni: that Harvey illicitly pressed unlike things together: psychology and physiology.^17^ Goldberg notes that analogies are *supposed* to pair unalike things.^18^ In this way, like Giglioni, Goldberg also assumes a radical distinction of ‘psychological’ and ‘organic’ or physiological categories. Relatedly, in Lennox’s view, Harvey’s use of Aristotle’s theory of forms does not work, because he obscures his own investigation by ‘relying on metaphors of conscious intention to clarify natural teleology’.^19^ For all these scholars, despite the differing shades and weights of their assessments, Harvey’s analogy problematically presses intentionality together with natural process. In these critical estimations, ‘animal’ and ‘natural’ conceptions, to use Harvey’s terms, are not the same or even similar.

The accusation from Goldberg, Giglioni, and Lennox here is that Harvey falls into the trap that, from his own time, Aristotelian teleology had always been understood to be: that it was animistic or anthropomorphising.^20^ As Steven Shapin and others have described, the ‘human-scaled’ Aristotelian accounts of nature were falling out of fashion, being replaced by ‘mechanical philosophy’, so-called because it compared nature, not to the rational human in the way that Aristotle did, but to the machine. Amongst Harvey’s contemporaries, then, purpose and intention were not usually, in correct accounts, assigned to natural entities. Yet, just as microscopes have gained currency since Harvey, so has the unconscious. In his example, Harvey is not offering animist motivations or sentience to stones or plants; rather, he is concerned with the animal, and often the human animal. Furthermore, whilst of course modern scientists use microscopes, I suggest that lay bodily experience, whether modern or early modern, is actually more closely aligned with Harvey’s findings. The interior of the uterus is still not visible to the eye outside of the clinic, and women cannot dissect themselves, like one of Harvey’s chickens or deer, and deploy the microscope technology that he overlooked. That Harvey, like everyone else, was bound by available knowledge and supplied terms is an undeniable fact; indeed, Harvey himself hopes that: ‘whatsoever falleth from me concerning this subject, I desire may not be so taken, as if I conceived them pronounced by an *Oracle*’ (539). As Noga Arikha says of Harvey’s loyalty to the increasingly unfashionable Aristotle: ‘[r]ather than dismiss his predecessors, Harvey looked anew, and found that the past could be used’.^21^ Approaching it from this perspective, I ask: what can be used from Harvey’s *De conceptione*, and particularly to consider false pregnancy?

What goes unnoticed in modern historians’ rejections of Harvey’s brain-uterus analogy is that Harvey is not only and principally interested in generation. Rather, he is concerned with conception and, for Harvey, generation and conception are different things. Indeed, *De conceptione* begins and is motivated by non-generation and, in particular, the perplexing issue of false pregnancy:

For though the *female* sometimes (conceiving after *coition* [*post coitum, concipiens*]) doth not produce a *Foetus*: yet we know that those *Symptomes* did ensue, which gave a cleare testimony [*claram fidem*] of a *conception* set on foot [*peractae*], (though it came to nothing.) (540; Latin 294)

The first parenthesis makes the case unequivocally: it is possible to have conceived even in the absence of a foetus. The next clause reiterates: Harvey’s knowledge comes from the appearance of symptoms which produce a clear promise or guarantee (*fidem*) of a conception achieved (*peractae*). In his view, false pregnancies are conceptions. Because he cannot see continuous matter, Harvey concludes that: ‘[w]e have no refuge left us, but to fly to meere *Conception* [*merum conceptum*], and *reception* of *Species* without any matter [*speciumque sine materia receptionem*]’ (547; Latin 297). Latin *merum* carries a sense of purity and is not perfectly translated by modern English ‘mere’, insignificant or feeble. Of course this is what Harvey proposes for all conceptions, fruitful or not, yet the idea of the ‘mere conception’ enables him to include non-generative conceptions in his understanding of what happens inside the body after sex. To read it in the terms of Harvey’s own analogy, an artist might conceive an idea, suffering the pangs attendant on artistic process, but, then, not generate the work. Sex generates expectations which may not always deliver.

False pregnancy was a significant concern in Harvey’s time, being fully discussed in a number of culturally apparent places. This is shown, for example, by Jonathan Gil Harris in his discussion of pregnancy and dropsy, both on the Renaissance stage and in the political debates concerning the childlessness of queens Mary and Elizabeth.^22^ A range of different conditions resembled, and were read as pregnancy: Harris particularly discusses dropsy or windy tympany, which swelled the abdomen. Cathy McClive has discussed other possible confusions, for example around molar pregnancy, and also made the larger point that all pregnancies were ambiguous at least until quickening.^23^ The discussion of false pregnancy also recognised that sometimes there was no identifiable organic cause or lesion. In these cases psyche and soma seemed mutually bound, creating the symptoms of pregnancy even in the absence of any embryonic or extraembryonic tissue. Negative diagnoses were likely more difficult to establish than positives, producing longer phases of ambiguity in the case of those suffering with infertility. When the midwife Jane Sharp discusses false pregnancy she prevaricates, writing that: ‘[t]o distinguish then false conceptions from true, but if there be both true and false at once that is very hard to know’.^24^ Although she writes as if ‘true’ and ‘false’ conceptions can be differentiated, giving them different and contrary labels, at the same time they are hard to tell apart, unless, and this is baffling, the conception is both true and false at once. Her statement is circular, maybe even a joke: indeterminate diagnoses are the only ones available and yet can only be accurate if the pregnancy is itself actually somehow indeterminate. Sharp’s indecision is revealing about the very difficult diagnostic frame in which Harvey’s investigations sit.

The psychophysiology or intelligence of early modern bodies and, more specifically, Harvey’s thinking about a brain-like uterus, offer a way in to thinking about the confounding case of the somatoform kind of false pregnancy. To begin this task, I return to Harvey’s concern with the artistic brain and ‘animal conception’. Harvey considers a spectrum of ‘animal conceptions’ and not just the one I’ve already discussed – the case of the artist producing life-like depictions or sculptural forms. First, he notes, birds ‘which immure themselves all winter, do exactly chant, and recall to minde those Ditties, the next Spring, which they had learned the Summer before, though they did never practise them all the while’; secondly, he marvels at birds that can build nests, and spiders that can spin webs exactly similar to others that they have never seen, ‘and that not from her memory, or any habit implanted in her, but onely by meere phansie’ [*sola phantasia*] (545; Latin 296). Harvey’s point is, again, about the immaterial connection, the gap between copies and originals, arranging his examples as more or less ‘strange’ according to how far they are mediated by a discernible or material pattern. Harvey’s view of fiction here is that facticity emerges from immaterial reproduction: that ‘meere phansie’ can bring into being a nest, a web, a song.

By framing animals and humans together, Harvey also explores a spectrum of ‘animal conception’ which ranges from conscious, cerebral acts to involuntary and instinctual ones which already look a great deal like the ‘natural conceptions’ that occur in the womb. In this way, ‘animal conception’ also opens up a question for Harvey about the nervous system: not only about how the uterus is like the fiction-making brain but also about how the brain, operating beyond as well as within consciousness, is like the uterus: conceiving of things without being aware of itself as engaged in a process of thought. In his 1627 manuscript work on the nervous system, *De motu locali animalium*, Harvey marks out the uterus as being like other organs outside conscious control, like ‘separate living creature(s)’, such as the heart, and the intestines, contrasting them with those that were wholly or partially subject to the will, like the muscles or lungs.^25^ Whilst, of course, the heart is different to the uterus and other organs of the autonomic nervous system, nonetheless it offers a model which unseats cerebral priority. In these notes on nerves, Harvey also thinks about bodily processes: the release of semen and childbirth are not subject to the will, whereas swallowing and, partially, vomiting are.^26^ He complicates this picture of the relation between consciousness and movement, however, by conceding that the voluntary nervous system might operate even when we forget, or pay no attention to it.^27^ Harvey also recognises that ideas can produce involuntary physical change: people shudder at fearful thoughts, for example, and their ‘parched mouths run a water at talke of sower thinges’.^28^ Quite apart from his work on the uterus and generation, Harvey asks how thoughts and passions can manifest themselves somatically, independently of conscious will.

In his assessment of Harvey’s uterus-brain analogy, Giglioni, asks: ‘are the transfers of meaning between the reproductive and the nervous systems at their end with Harvey?’, to which he expects the answer: yes, finding that biochemistry and genetics have dismantled earlier metaphoric thought-structures which made that transfer.^29^ However, we can answer this question differently by turning to the work of feminist Elizabeth A. Wilson, who finds that the nervous system is indeed implicated in the workings of the whole body, a body which includes the reproductive system. In her book *Psychosomatic*, Wilson challenges the resistance to biology in contemporary feminism, in order to recognise the role of the body, as well as the encultured mind, in psychosomatic conditions. In doing so she recommends a recovery of pre-disciplinary accounts of the relation of mind and body which present them as together and indivisible. Harvey offers ways to think about somatoform conditions, which, as Wilson argues, require the participation of both brain and periphery. Psychology, or a ‘proclivity to conversion’, Wilson concludes, is ‘native to biochemical, physiological, and nervous systems’.^30^ In this way, Wilson maintains that puzzling relationships between soma and psyche are ‘not just in the head’; that *psychological* responses are produced at the place they are felt. A paralysed arm, for example, can manifest bereavement because the arm itself thinks. In this view, the body is intelligent in the way that Charalampous notes early modern commentators always imagined.^31^ That the uterus functions like, or in exactly the same way as a brain, as Harvey suggests, is exactly what Wilson offers: the body as ‘a system of mutual constitution from which no particular element emerges as the originary, predetermining term’.^32^

Wilson has suggested indeed that we reclaim the older, Hippocratic notion of the errant womb as a potentially useful figure encapsulating a truth about all organs, male and female: that they are vagrant. Perhaps, she argues, ‘all biology wanders. Formulated this way, hysterical diversion is not forced on the throat, legs, or eyes from the outside, it is already part of the natural repertoire of biological matter’.^33^ Wilson offers to Harvey a model of how a passion, like love, might be biologically implicated at a site, like the uterus, beyond the conscious or unconscious governance of the brain. Harvey offers Wilson a number of related advances on her reclamation exercise. First, although in his writing on generation Harvey is necessarily principally concerned with the uterus, the psychophysiology which underpins his thinking, already de-particularises that organ, understanding it to be psychologically entangled only in the same way that other organs are, and those potentially of both men and women. For Harvey hysteria and other uterine pathologies are clearly different and not the condition of everywoman. Thus he sees that the entwinedness of body and mind can be part of a picture with health as much as illness. Finally, Harvey supplies a ready way of considering particularly non-reproductive experience in relation to psychophysiology.

The proximity, similarity or interchangeability between body and brain allows us to see how, for Harvey, passions, like love and desire, not only motivate one human animal to move towards another but, before that, how passions cause motion within the biology of that animal. In what follows I look more closely at how different forms of love drive distinctions between uterine function and dysfunction, considering non-generation, as Harvey did, in relation to fictions of the wind. In particular, throughout his work on generation, Harvey takes a special interest in wind eggs. They are variously referred to by the Latin terms ‘improlifica, irrita, hypenemia, sive subventanea, & Zephyria’ (37), the last three of these register, as does the English ‘wind egg’, the perception that ‘they were begotten by the winde’ (68). The relationship to the wind enables an agnosticism – wind was a sign of both vacancy and of life, of flatulence and breath – an agnosticism which was useful given the difficulties of diagnosis acknowledged by Sharp. This agnosticism had blown from the ancient world and through the Middle Ages. Early pregnancy was hard to diagnose in just the same way that wind eggs looked like fruitful ones. Birds’ wind eggs had been a concern and curiosity since Aristotle, and were regularly discussed in encyclopaedias for example, throughout the Middle Ages.^34^ As we have seen, windy dropsy was a condition in which wind swelled the abdomen in a way which resembled pregnancy.^35^ Yet equally, wind, as divine breath, inspired and ensouled the quickening foetus in all pregnancies, and was invoked to explain the exceptional case of the Virgin conception.^36^ From the ancient to the Renaissance world, the microcosmic body was in ecological relation to the larger macrocosm; wind and water blew and flowed through people, connecting their bodies and passions, in close combination, to the cosmos.^37^ Wind, then, refused human exceptionalism, making no distinction between people and the natural world. All life was animated by wind but, equally, eggs that did not hatch and apparent pregnancies which did not deliver, hollow rounded-out forms, were put down to the wind.

Using the ideas he inherited, Harvey draws specific conclusions about human non-generation from his observation of birds’ wind eggs. He notes in *De motu locali*: ‘homo enim the text, the other the comment’ [for man is the text and the other, i.e. the animal, is the commentary], unthinkingly code-switching just at the point when he meets the human/animal species barrier.^38^ In the case of conception, the exteriority of birds’ eggs offer a legible commentary on the resistant interiority of the human reproductive text. Yet the wind egg, more specifically, gives Harvey a way to think about different kinds of non-generation. In poultry rearing, when hens are kept from the cock, Harvey finds they ‘not onely *conceive egges*, but lay them also’ [*non solum ova concipit, sed & parit etiam*] (27; Latin 15). So, at one end of the spectrum, cyclical signs of fertility and particularly menstruation are understood by comparison to wind eggs. Wind eggs are produced even without intercourse, part of the regular round of the reproductive cycle. Although Harvey uses the verb *concipere* here, he makes a clear distinction between eggs and conceptions (between the nouns *conceptus* and *conceptio*), throughout his work. *De conceptione* earns its title by being concerned with conceptions proper; because *De conceptione* is principally presented as a solution to the puzzle of the missing spermatic mass, it is necessarily only concerned with post-coital events. At the other end of the spectrum from menstruation and other cyclical signs of fertility, Harvey likens wind eggs to non-generative conceptions after intercourse:

Divers Women, whose *Conception* (like an addle *Egge*) is fruitles, and without a *Foetus*, do suffer *abortion* the third moneth. I have often dissected an *abortion* of that *age* (being of the bigness of a *Goose-egg*) wherein was a *foetus*, distinct in all its parts; though their form was rough and unshapen’. (335–6)

Here Harvey is concerned with first-trimester pregnancy loss, where the miscarried conceptus might possibly be with or without foetus. From menstruation and early miscarriage, to false pregnancy and conditions like dropsy which resemble pregnancy, the wind egg is analogous to the multiple ways in which generative potential is not realised.

Despite this wide range of ways of understanding the human wind egg, for Harvey, sex or lack of it makes the difference, separating out conceptions – whether with or without a foetus – from eggs. Blocked or satisfied erotic desire acts on the body and changes it, even though it may not result in offspring. Although in *De generatione* Harvey discusses the way that birds which are not mated try to incubate unfertilised eggs, in *De conceptione* when he discusses birds inclined to sit on wind eggs, and also false pregnancy in dogs, he is careful to add a clause about coitus. Bitches, he says, that ‘admit coition’ can ‘have *milk*, or *beestings* (as they call it) in their teats; and are obnoxious to the distempers of those that have really *puppied’* (540). Similarly birds, as dogs, present as if they have reproduced although they haven’t, building and sitting on their nests:

Some kinde of *birds* (as namely *Pigeons*) if they admit *coitione* at the wonted time, though they lay no eggs at all, or *subventaneous* ones onely, yet are possessed with their usual sedulity & providence of *building nests*. (541)

This emphasis upon intercourse enables Harvey to disambiguate the animals he considers here from the unmated ones he discusses in *De generatione*: in their case, after sex, maternal habitus marks out a conception achieved. Harvey’s observation of maternal behaviours in birds makes little distinction between those whose eggs hatch, and those whose eggs do not. Because good eggs cannot be told from bad except in time, Harvey uses affective contexts – desire; sex, both practised and inhibited; and maternal devotion or attention – to distinguish them. Whilst Thomas Laqueur cites the objection that, if Harvey’s account ‘were true, women should be able to conceive by just thinking about it’, Harvey might reply that women may well be able to conceive, first by having sexual intercourse and then afterwards by ‘thinking’ about it.^39^

Unsurprisingly, whether or not love and desire are expressed through the sexual act is important in relation to reproduction; what is less obvious is that sexual expression is also biologically momentous in the case of non-generation. Harvey takes the contemporary view that love-sick females who are held back from a desired sexual life become ill. In *De generatione* Harvey says he discovers this by observing his wife Elizabeth’s parrot. This is an animal that he assumed was male, because it was so vocal and tuneful, an aspect of the amorousness it displays in its relationship with Elizabeth (24–5). However, during the autopsy Harvey performs on the parrot after its death, he discovers it to be female and to have a corrupted egg retained in its uterus. He imagines that something similar can happen to girls:

As if it were the same thing for those creatures [ie some insects and hens] to be with egge, as for *virgins* to have their *wombs grow warm*; their *termes flow*, their *breasts increase*, and (in a word) to become *marriageable* [*viro maturam esse*]; which if they be too long detained from, they are assaulted with dangerous *symptoms*; (namely *hysterical affections*, or *furor Uterinus*) or else fall into the *green sickness*, and severall other distempers. For all Creatures [*animalium*], when they are love-struck [*cupidinis oestro percita*], grow extravagant, and debarred of enjoyment [*nisi se invicem fruantur*], do at length recede much from their usual *temper*. Hence some women grow frantick for love; and this extravagancy is so *outragious* in some, that they seem *bewitched* [*venesicio afflatae*], *planet-struck* or *possessed*. (27–8; Latin 16)

In his discussion of hysterical affections and related conditions here, Harvey is not concerned with conception. Instead he charts what we might call sexual repression as a cause of female sickness. In that sense, Harvey recognises non-generative conception to be distinct from hysteria and associated conditions, not a pathology but a relatively ordinary consequence of a sexual life. Harvey’s narrow understanding of hysteria and related conditions, then, does not fit the picture of ‘Hysteria […] before 1750’, described by Heather Meek as suffered by ‘all women’ ‘to some degree’.^40^

Harvey borrows the phrase ‘cupidinis oestro percita’ [literally: stirred by the gadfly of desire], in the quotation above, from Robert Burton’s *Anatomy of Melancholy*, where the *oestrum*, or bite of the gadfly, is evoked to retell the story of Sappho and Phaon.^41^ This entomological image, which implicitly casts people as cattle, offers a suggestive metaphor, as animals often do for Harvey, for how desire moves and changes the human body quite involuntarily and beyond the reach of reason. Indeed, erotic impulses are examples of some of the ‘inward principles’ – ‘desire, imagination, choice, wish, passion, appetite’ – which he lists in *De motu locali animalium* as causing movement within animals.^42^ Harvey’s appropriation of Burton betrays a wider engagement with his work. Rather than using those bits of the *Anatomy* that were particularly concerned with uterine and menstrual health, Harvey instead cites and engages with Burton’s definitions and understanding of love sickness.^43^ In this respect Harvey resorts to the contemporary correlation, which Helen King and Lesel Dawson have discussed, between female love sickness and gynaecological diagnoses, and particularly green sickness.^44^ Dawson has also argued that early modern love sickness implicated the imagination: ‘[t]he lover dotes obsessively, not on the true physical form of the beloved, but on the *phantasm*: the perceived, spiritual image that is impressed upon his or her mind’.^45^ Indeed, traditionally in earlier medieval love literature the image of the beloved travelled into the lover’s heart through the eye, through looking.^46^ By signing up to the associations between love sickness and gynaecological pathology that emerged later to embellish this relation between love and looking, Harvey produces a different account of how the female mind relates to the reproductive body in illness as opposed to health. Frustrated desire, in love sickness, causes a pathological or corrupted animal conception, whereas natural conceptions, whether generative or not, are corollaries of a sexual life.

In the story of Elizabeth’s parrot, Harvey brings the question of the difference between un-generative conception and uterine pathologies home. Elizabeth Spiller has also discussed this parrot in her satisfying account of Harvey’s writing on generation.^47^ Spiller’s larger argument is that Harvey is as, if not more, concerned with his own act of creation, his writing, as he is with reproduction more broadly, noting his emphasis on ‘fiction, art and fable’.^48^ Harvey, she writes, ‘is talking not so much about women, hens, or does as about himself and his acts of scientific creation’.^49^ Spiller, though, sees Harvey’s preoccupation as being with ‘pregnancy and parturition’, to fit with this reflexive interest in literary creation.^50^ Yet, as I have been arguing, Harvey is at least as interested in non-generation, and particularly so in the anecdote about the parrot. The account of Elizabeth’s parrot stands out for its autobiographical interjection – this is the only place across his oeuvre that Harvey mentions his wife – and what does it describe? Spiller suggests that Harvey’s autopsy finds the bird to be ‘morbidly pregnant’; but that is not so, as the bird was never mated.^51^ After all, what is pregnancy in birds? As Harvey himself notes, the avian foetus gestates in the egg in the nest, rather than internally in the bird’s uterus (*De generatione*, 4–5). Rather, Elizabeth’s queer parrot, gendered masculine but anatomically female, is love sick, green sick, or a hysteric, its putrefying egg like retained menses. The story shortly precedes Harvey’s concerns about marriageable girls suffering ‘hysterical affections’ that I discussed above. Harvey’s autopsy of the parrot is an investigation not into pregnancy but rather female love-sickness, and enables him to sort health from disease in the case of non-generation.

Harvey’s description of the parrot’s attempts to court his wife are full and excessive, charting its ‘mutterings’ and ‘shaking of his wings’, its ‘familiarity’ and ‘obsequiousness’, its ‘singing and talking’ (24). The parrot dies, Harvey says, in Elizabeth’s lap or bosom – his Latin word is *gremium* – which he also uses earlier to denote the place the parrot liked to lay its head to be stroked when it was alive. He complains that in life the animal ‘was now grown so familiar, that he was permitted to walk at liberty through the whole house’ (24), taking up an illegitimately intimate place in his marital home, even in his wife’s lap. Keller has considered Harvey’s descriptions of himself throughout his writing on generation and conception as a ‘torchbearer’ and ‘hero-adventurer’.^52^ Yet, in her concluding comments, she finds that Harvey’s ‘images of masculine triumph […] ultimately appear hollow, generated, like the embryo itself, on a perceived absence’.^53^ In his resentment of Elizabeth’s parrot, Harvey offers something much more like that which Keller describes in this conclusion. Any account of Harvey’s own marriage or amatory feelings is displaced by his experience of this eccentric love triangle. Indeed, in the competition for Elizabeth’s love, rather than being preferred to the parrot, Harvey only wins out by outliving and dissecting it. Necessarily the relationship between Elizabeth and the parrot – a bird which, anyway, turns out to be female – is un-generative, being impossible, and, in that respect, Harvey chooses a curious love-rival against which to measure himself.

The narrative of the parrot’s love is interrupted by a digression, derived from Aristotle and, again, Virgil’s *Georgics*, on birds and windy reproduction. Harvey says he reads in Aristotle that male birdsong can be carried on the wind and fecundate a hen bird:

*Aristotle* saith, *If partridge-hennes stand over against the cocks, and the winde blow from whence the cocks are, they conceive and grow big, and for the most part, they teem* [*ingravescunt*] *even by the voice of the cock, if they be at that time wanton and lustfull* (24)

Spiller sees in the discussion of birdsong the figure of the potent male artist and, indeed, of Harvey himself.^54^ Yet what are described here are not necessarily fruitful conceptions. Harvey does not specify whether these are conceptions with or without foetuses. Harvey’s Latin is more equivocal than the English; the word *ingravescunt* [grow heavy] does not populate the abdomen in the same way that English ‘teem’ does. Abdomens may swell and gain weight, but not gestate or deliver young. Tellingly the lengthy quotation from the *Georgics* which follows describes the fructifying descent of the god Aether into the joyful womb, lap, or bosom of his consort: the earth. The Latin phrase is ‘*gremium laetae*’, (Latin 14; English 25); and, as I noted, Harvey uses the same word, *gremium*, twice elsewhere in his description of Elizabeth’s intimate interactions with her pet. Whilst Harvey’s anecdote about the parrot at first appears to be about the parrot’s un-generative pathology, in the repetition of *gremium*, it also elliptically points out Elizabeth’s un-generativeness, and a womb, which Harvey’s biography reminds us, did not bear children.^55^ In this triangulation, Harvey’s own childless marriage is offered up in implicit contrast to the chaste but similarly un-reproductive love that the parrot holds for Elizabeth. If animals are the commentary which make explicit what is implicit in the human text, the parrot speaks eloquently, if obliquely, about Harvey’s personal interest in non-generation.

Elizabeth’s frustrated parrot also stands in contradistinction to those broody birds that Harvey returns to throughout his writing on generation, who incubate subventaneous or addled eggs. Pathological love-sickness, like the parrot’s, is not conception, unlike the wind eggs of mated birds. In this way, Harvey credits false pregnancy and recognises that it provokes or is accompanied by maternal behaviours in animals, which he observes with some tenderness. For all that Keller is right on Harvey’s ‘sex stories’, and Spiller, too, on his generative fictions, we should also acknowledge Harvey’s insight into non-generation. This is particularly evident at the moments when Harvey self-interrupts with literary quotation particularly from the *Georgics*; fiction offers the best, because most immaterial, grounds for approaching non-generation. However, Harvey’s use of Virgil’s poem is characterised by resistance to its compulsory reproductive logic. In particular, Harvey is careful not to exclude un-generative wombs, and particularly not Elizabeth’s, from his version of the erotic descent of Aether. If Virgil had used the spring winds to link eros and reproduction, Harvey instead finds this erotic moment in Virgil’s text useful to consider not infertility exactly but rather an in-between diagnostic state which may be generative, but equally may not. Harvey understands that conceptūs without foetuses have seasons, just in the way that fruitful ones do, that there are particular times when expectations rise. Harvey replaces Virgil’s emphasis on reproduction with another, on conception, and additionally offers maternal affection, which is not a concern in Virgil’s poem, as a sign of a conception achieved after intercourse. In this way, he dignifies the un-generative body by including it in ancient erotic dramas which placed human sexuality in cosmological and natural perspective. Furthermore he reorients his readers to consider birds that sit on wind eggs – and perhaps the falsely pregnant women they resemble – and to acknowledge their loves, desires and maternal ministrations, as much as those whose sitting is rewarded with ‘chickens to discipline […] assemble, nurture, feed and protect’ (71).

Whilst many critics have dismissed *De conceptione* as itself un-generative and limited by its place in history, to his credit, Harvey recognises and is reflective about his partial perspective. Unable to see the sperm ‘remaining behinde in the female’ (548), he draws this blank:

What remains, since I can imagine nothing else, nor no man hath hitherto dreamed of any other thing, but freely to profess my self to be at a stand? (548)

As Spiller has argued, Harvey’s work on generation and particularly *De conceptione* is concerned with his own act of creation; this passage is no exception.^56^ The question of the immaterial remnant links up Harvey’s fallacious observation of the material gap in conception to his confession about his own limitations. Harvey anticipates that new light will come in, as indeed it has, asserting that he contributes only a ‘conjecture’ until ‘there be some certainty established in the business’ (547). His emphasis upon the imagination in the quotation here demonstrates that his contribution is itself a conception in the terms set out in his analogy: one that may or may not prove fruitful, that exists in a diagnostic in-between only to be resolved by time. He insists, though, that his is no ‘monstrous matter’ (546), using the lexicon of teratology to defend his conception from a charge of deformity. Harvey anticipated the bad press his analogy would receive: ‘I know full well, that some scoffing persons will laugh at these conjectures’ (546), he says, but defends his contribution on the grounds that all opinions begin as ‘meere figments, and imaginations’ [*mera figmenta & imaginationes*] (546; Latin 297).^57^ His use of *merum* here apparently empties fiction of its value except that he deploys the same word in his awed account of the body’s powerful creative capacities. In *De conceptione* Harvey finds that conceptions are ‘mere’ works of the imagination, yet they can be credited, even in the absence of a foetus, not only because they are accompanied by biological symptoms of pregnancy, but also because those symptoms are generated by erotic and romantic expectations and signed by maternal love. Love is crucial to Harvey’s symptomology of the intelligent un-reproductive female body, and of a womb that can envisage, although perhaps not always deliver, the children often looked for in a sexual life.

## Notes

1. I take this term from Gail Kern Paster, *Humoring the Body: Emotions and the Shakespearean Stage* (Chicago: University of Chicago Press, 2004), p. 12. Throughout this essay I primarily cite the first English translation of Harvey’s work, mostly discussing the appendix *De conceptione*, but also considering the more substantial work which precedes this and other appendices, referring to it as *De generatione*: William Harvey, *Anatomical Exercitations Concerning the Generation of Living Creatures* (London, 1653) H1085 (Wing). For brevity’s sake I include Harvey’s Latin selectively; I use: William Harvey, *Exercitationes de generatione animalium* (London, 1651) H1091 (Wing). Hereafter I cite these texts by page number in parenthesis in the text.

2. The quotations here come from Benjamin Goldberg, ‘A Dark Business, Full of Shadows: Analogy and Theology in William Harvey’, *Studies in History and Philosophy of Biological and Biomedical Sciences*, 44 (2013), pp. 419–32 (pp. 431 and 419). I discuss this and other responses below.

3. See, for example, Thomas Laqueur, *Making Sex: Body and Gender from the Greeks to Freud* (Cambridge MA: Harvard University Press, 1990), pp. 143 and 146; Guido Giglioni, ‘*Conceptus uteri / Conceptus cerebri*, note sull’analogia del conceptimento nella teroia della generazione di William Harvey’, *Rivista di storia della filosofia*, 48 (1993), pp. 7–22 (p. 8).

4. Elizabeth A. Wilson, *Psychosomatic: Feminism and the Neurological Body* (Durham NC: Duke University Press, 2004).

5. Charis Charalampous, *Rethinking the Mind-Body Relationship in Early Modern Literature, Philosophy and Medicine: The Renaissance of the Body* (London: Routledge, 2015), see esp. p. 2.

6. Virgil, *Eclogues, Georgics, Aeneid I-VI*, trans. H. R. Fairclough, revised G.P. Goold (Cambridge MA: Harvard University Press, 1999), *Georgics*, II, p. 159. Fairclough’s translation has been chosen, over a translation more contemporary to Harvey, to get closer to the literal sense of the cited Latin; it has also been reordered to mirror Harvey’s own reordering.

7. Paster, *Humoring the Body*, p. 9.

8. Eve Keller, ‘Making up for Losses: The Workings of Gender in William Harvey’s *de Generatione animalium*’, in Susan C. Greenfield and Carol Barash (eds), *Inventing Maternity: Politics, Science, and Literature, 1650–1865* (Lexington: University Press of Kentucky, 1999), pp. 34–56 (p. 43).

9. Ibid., p. 44.

10. See also James G. Lennox, ‘The Comparative Study of Animal Development: William Harvey’s Aristotelianism’, in Justin E. H. Smith (ed.), *The Problem of Animal Generation in Early Modern Philosophy* (Cambridge: Cambridge University Press, 2006), pp. 21–46 (p. 38).

11. On the traditional medieval gendering of hylomorphism, see Kellie Robertson, ‘Materiality and the Hylomorphic Imagination’, in Holly Crocker and D. Vance Smith (eds), *Medieval Literature: Criticism and Debates* (London: Routledge, 2014), pp. 367–75 (esp. 368–9).

12. Keller, ‘Making up for Losses’, p. 53; Lennox, ‘The Comparative Study of Animal Development’, p. 38.

13. For accounts of the maternal imagination see, for example: Jenifer Buckley, *Gender, Pregnancy and Power in Eighteenth-Century Literature: The Maternal Imagination* (London: Palgrave Macmillan, 2017) and Marie-Hélène Huet, *Monstrous Imagination* (Cambridge MA: Harvard University Press, 1993).

14. See, for example, Justin E. H. Smith, ‘Introduction’, in Smith (ed.), *The Problem of Animal Generation*, pp. 1–18 (p. 10).

15. Goldberg, ‘A Dark Business’, the quotation is from p. 428, but see also p. 425 and 431.

16. Ibid., p. 419.

17. Ibid., p. 427, responding to Giglioni, ‘*Conceptus uteri / Conceptus cerebri*’, p. 22.

18. Goldberg, ‘A Dark Business’, p. 427.

19. Lennox, ‘The Comparative Study of Animal Development’, p. 45. See, also, Goldberg, ‘A Dark Business’, p. 423.

20. Steven Shapin, *The Scientific Revolution* (Chicago: University of Chicago Press, 1996), pp. 28–30.

21. Noga Arikha, *Passions and Tempers: A History of the Humours* (New York: Harper, 2007), p. 182.

22. Jonathan Gil Harris, ‘All Swell That End Swell: Dropsy, Phantom Pregnancy, and the Sound of Deconception in *All’s Well that Ends Well*’, *Renaissance Drama*, 35 (2006), pp. 169–89 (*passim*). See also the work done on Mary Tudor, e.g. Judith M. Richards, *Mary Tudor* (London: Routledge, 2008), pp. 173 and 178.

23. Cathy McClive, ‘Hidden Truths of the Belly: The Uncertainties of Pregnancy in Early Modern Europe’, *Social History of Medicine*, 15 (2002), pp. 209–27 (esp. pp. 214–22).

24. Jane Sharp, *The Midwives Book: Or the Whole Art of Midwifry Discovered*, ed. Elaine Hobby (Oxford: Oxford University Press, 1999), p. 88.

25. William Harvey, *De motu locali animalium*, ed. and trans. Gwyneth Whitteridge (Cambridge: Cambridge University Press, 1959), pp. 110–11.

26. Ibid., pp. 40–1.

27. Ibid., pp. 122–3.

28. Ibid., p. 98.

29. Giglioni, ‘*Conceptus uteri / Conceptus cerebri*’, p. 22: ‘sono poi davvero terminati con Harvey i trasferimenti di senso tra sistema riproduttivo e sistema nervoso?’.

30. Wilson, *Psychosomatic*, p. 13.

31. Charalampous, *Rethinking the Mind-Body Relationship*, eg. p. 2.

32. Wilson, *Psychosomatic*, p. 19.

33. Ibid., p. 13.

34. Bartholomaeus Anglicus, *De proprietatibus rerum*, trans. John Trevisa, STC 2nd edn (London, 1537), Book XIX, ca 79, Sig. q vi verso– r verso. See, also, Maaike van der Lugt, *Le ver, le démon et la vierge: les théories médiévales de la génération extraordinaire* (Paris: Les Belles Lettres, 2004), pp. 123–4.

35. See for a full discussion, Sally Frampton, ‘The Debris of Life: Diseased Ovaries in Eighteenth-Century Medicine’, in Raymond Stephanson and Darren N. Wagner (eds), *The Secrets of Generation: Reproduction in the Long Eighteenth Century* (Toronto: University of Toronto Press, 2015), pp. 344–63 (pp. 350–3).

36. See Margaret Berger ed. and trans., *Hildegard of Bingen: On Natural Philosophy and Medicine. Selections from Cause et Cure* (D. S. Brewer: Cambridge, 1999), e.g. p. 45. See also, Katherine M. Kueny, *Conceiving Identities: Maternity in Medieval Muslim Discourse and Practice* (New York: SUNY Press, 2013), pp. 204–6.

37. Paster, *Humoring the Body*, p. 19; Arikha, *Passions and Tempers*, p. 3.

38. Harvey, *De motu locali animalium*, pp. 50–1.

39. Laqueur, *Making Sex*, p. 144.

40. Heather Meek, ‘Motherhood, Hysteria, and the Eighteenth-Century Woman Writer’, in Stephanson and Wagner (eds), *The Secrets of Generation*, pp. 238–57 (p. 239).

41. Robert Burton, *The Anatomy of Melancholy*, ed. Holbrook Jackson (New York: New York Review of Books, 2001) Part 3, section 2, member 5, subsection 4, p. 227. Burton’s was also a borrowing, he cites Menander, but more likely receives it from Theodor Zwinger, *Theatrum humane vitae* (Basel, 1586), Liber I, p. 18.

42. Harvey, *De motu locali animalium*, pp. 34–5

43. For Burton’s discussion of women’s melancholy, see *Anatomy of Melancholy*, Part I, section 3, member 3, subsection 4, pp. 414–19. See also, e.g. Paster, *Humoring the Body*, pp. 95–100.

44. Helen King, *The Disease of Virgins: Green Sickness, Chlorosis and the Problems of Puberty* (London: Routledge, 2004), pp. 36–42 and Lesel Dawson, *Love Sickness and Gender in Early Modern English Literature* (Oxford: Oxford University Press, 2008), esp. pp. 51–3.

45. Ibid., p. 23.

46. See, for example, Geoffrey Chaucer, *Troilus and Criseyde*, Book I, 295–8.

47. Elizabeth Spiller, *Science, Reading, and Renaissance Literature: The Art of Making Knowledge, 1580–1670* (Cambridge: Cambridge University Press, 2004), pp. 94–5.

48. Ibid., p. 90.

49. Ibid., p. 91.

50. Ibid., p. 92.

51. Ibid., p. 94–5.

52. Keller, ‘Making up for Losses’, pp. 51 and 53.

53. Ibid., p. 53.

54. Spiller, *Science, Reading and Renaissance Literature*, p. 95.

55. Roger French, ‘Harvey, William (1578–1657), physician and discoverer of the circulation of the blood’, *Oxford Dictionary of National Biography*. 2004-09-23. Oxford University Press. [date accessed 28 March 2018].

56. Spiller, *Science, Reading and Renaissance Literature*, esp. e.g. p. 91.

57. For an account of the objections Harvey’s work on generation received in its own time, see Ibid., p. 98. 

